# Coherent narrowband light source for ultrafast photoelectron spectroscopy in the 17–31 eV photon energy range

**DOI:** 10.1063/1.5131216

**Published:** 2020-01-31

**Authors:** Riccardo Cucini, Tommaso Pincelli, Giancarlo Panaccione, Damir Kopic, Fabio Frassetto, Paolo Miotti, Gian Marco Pierantozzi, Simone Peli, Andrea Fondacaro, Aleksander De Luisa, Alessandro De Vita, Pietro Carrara, Damjan Krizmancic, Daniel T. Payne, Federico Salvador, Andrea Sterzi, Luca Poletto, Fulvio Parmigiani, Giorgio Rossi, Federico Cilento

**Affiliations:** 1 C.N.R.-I.O.M., Strada Statale 14, km 163.5, Trieste, Italy; 2 Elettra-Sincrotrone Trieste S.C.p.A., Strada Statale 14, km 163.5, Trieste, Italy; 3 Dipartimento di Fisica, Università degli Studi di Trieste, via A. Valerio 2, Trieste, Italy; 4 IFN-CNR, Via Trasea 7, Padova, Italy; 5 Dipartimento di Ingegneria dell'Informazione, Università di Padova, via Gradenigo 6/B, Padova, Italy; 6 Dipartimento di Fisica, Università di Milano, via Celoria 16, Milano, Italy; 7 International Faculty, University of Cologne, Albertus-Magnus-Platz, 50923 Cologne, Germany

## Abstract

Here, we report on a novel narrowband High Harmonic Generation (HHG) light source designed for ultrafast photoelectron spectroscopy (PES) on solids. Notably, at 16.9 eV photon energy, the harmonics bandwidth equals 19 meV. This result has been obtained by seeding the HHG process with 230 fs pulses at 515 nm. The ultimate energy resolution achieved on a polycrystalline Au sample at 40 K is ∼22 meV at 16.9 eV. These parameters set a new benchmark for narrowband HHG sources and have been obtained by varying the repetition rate up to 200 kHz and, consequently, mitigating the space charge, operating with $\approx 3\times 10^{7}$ electrons/s and $\approx 5\times 10^{8}$ photons/s. By comparing the harmonics bandwidth and the ultimate energy resolution with a pulse duration of ∼105 fs (as retrieved from time-resolved experiments on bismuth selenide), we demonstrate a new route for ultrafast space-charge-free PES experiments on solids close to transform-limit conditions.

## I. INTRODUCTION

The expanding quest for studying the physics of complex and quantum materials under non-equilibrium conditions has prompted the development of advanced ultrafast light sources in order to tailor specific excited states and probe their electronic transient properties. By high harmonic generation (HHG)^1–5^ light sources, as well as free electron laser (FEL), it is possible to obtain radiation pulses with photon energies extending from the extreme ultraviolet (EUV) to hard x-rays, with pulse durations ranging from sub-fs to sub-ps, and fully polarized light. However, while these sources are suitable for high peak brilliance experiments, they show severe limits for ultrafast photoelectron spectroscopy (PES) on solids, where the photon density in the light pulses and the light pulses repetition rate must be controlled in order to reduce spurious effects.

PES^6,7^ allows us to measure, under perturbative conditions, the spectral function resulting from the projection of the final electronic states on the initial states of the matter. In quantum and strongly correlated materials, the collective excitations and the quasi-particles interactions will affect the self-energy; hence, the PES spectral function is used, and its features unveil the effects of these many body interactions on the kinetic energy and momentum of the primary photoelectrons. By measuring the kinetic energy and the momentum of the primary electrons, the PES experiments can be extended to the reciprocal space, therefore, to the measure of the band structure, while another degree of freedom of the photoelectron can be observed by detecting its spin.

In the last two decades, angle-resolved (AR) PES and spin resolved (SP) ARPES have been extended to time domain in the sub-ps regime. These experiments require stable (in terms of energy, polarization, intensity) pulsed sources of photons with a pulse duration in the range 10–100 fs and with a variable peak brilliance and repetition rate. Hence, the number of photons, for a fixed focal spot on the sample, has to be such that the space charge effects are minimized, while compensating the limited photoelectron statistics by raising the pulses repetition rate as high as possible to provide the optimal signal-to-noise ratio.

A conceivable light source for time-resolved photoelectron spectroscopy should respond to the following characteristics: (i) provide 10^3^–10^4^ coherent photons per pulse in the energy range 6 eV–100 eV and with ∼100 fs time duration, in order to exploit favorable photoionization matrix elements, while covering the full Brillouin zone of all materials; (ii) variable pulse power, pulse duration, and repetition rate; (iii) tunability of the photon energy in a broad range;^8–13^ and (iv) repetition rate of several tens of kilohertz, up to megahertz,^14–27^ but compatible with the relaxation time of the excited states, in order to mitigate thermal effects on the sample.

In particular, space charge^28–30^ can heavily affect the primary photoelectron kinetic energy and trajectory, hence limiting the experimental resolution. This is the most challenging problem for time resolved PES experiments in the direct and reciprocal space (angle resolved PES, i.e., ARPES).

Here we report on a novel HHG beamline that lays down a new benchmark in terms of repetition rate (up to 200 kHz), photons per pulse (up to 10^6^), pulse duration (∼100 fs), and an overall energy resolution for time resolved PES of ∼22 meV, as measured on polycrystalline Au at 40 K. The main characteristics of this source are summarized in Table I. By comparing the parameters in Table I with those of equivalent setups in the literature,^5,14,16,18–20,26,31–34^ the HHG time resolved ARPES described herewith below meets the state of the art in the field.

**TABLE I.** Time and energy resolution performance: $\Delta E_{\;\text{exp}}$, experimental energy resolution, including optical elements broadening and analyzer resolution; the electrons and photons per second are provided for the space charge free condition allowing for the overall energy resolution of 22 meV (9 pA at 16.9 eV and 200 kHz); $\Delta t_{\;\text{exp}}$, experimental time resolution, measured as convolution between pump and probe pulses; $\Delta E_{\mathrm{probe}}$, harmonic bandwidth, after deconvolution of temperature and analyzer contribution; $\Delta t_{\mathrm{probe}}$ probe pulse time duration; $\Delta t_{FT}$, estimation of Fourier transform pulse duration, obtained considering a time-bandwidth product ≈ 0.44.

Parameter	Value
Photon energy (eV)	16.9
Rep. Rate (kHz)	200
$\Delta E_{\;\text{exp}}$ (meV)	22
Electrons/s	≈ 3 × 10^7^
Photons/s	≈ 5 × 10^8^
$\Delta t_{\;\text{exp}}$ (fs)	300
$\Delta E_{\mathrm{probe}}$ (meV)	19
$\Delta t_{\mathrm{probe}}$ (fs)	105
$\Delta t_{FT}$ (fs)	96

The key to allow for bright and narrowband harmonics generation was pointed out by Wang *et al.*^35^ and others,^19,36,37^ and consists in seeding the HHG process by Ultraviolet (UV) (≈ 3 eV) photon pulses. Here we extend this concept to the case of Yb-based laser sources and seed the HHG process at 2.4 eV (as also reported in Ref. 27). This method allows to obtain harmonics bandwidth of the order ≈ 20 meV, a value similar to what currently attainable by cavity-enhanced harmonics generation setups.^16,17^ However, in the latter case, the fixed and high repetition rate (60–88 MHz) puts severe constraints on pump-probe experiments, while Yb-base amplifiers allow for a straightforward and wide repetition rate tuning that allows to limit the pump beam average power. Although typically operating at a fixed repetition rate, Ti:Sapphire lasers allow to generate ≈ 60 meV bandwidth and <60 fs long harmonics for ARPES experiments with UV-driven HHG at 50 kHz (as reported in Ref. 14). Alternatively, for IR-driven high-harmonics in a hollow-fiber at 30 kHz (as reported in Ref. 26), the bandwidth must be filtered and reduced to allow for 30 meV resolution at the expense of a reduced flux and an increased pulse duration. Finally, UV-driven HHG from an optical parametric chirped pulse amplifier (OPCPA) high-power laser system has been demonstrated in Ref. 33. This approach combines the advantages of a high repetition rate operation (500 kHz), a high average flux, and a high time-resolution (<40 fs) while providing harmonics bandwidth of ≈ 110 meV.

## II. HHG SOURCE AND PHOTON BEAM OPTICS

### A. The laser source

The source is a Yb:KGW-based integrated femtosecond laser system (PHAROS, Light Conversion), characterized by a turn-key operation and by high pulse-to-pulse stability. The system produces ∼300 fs pulses at 1030 nm with a tunable repetition rate from single-shot to 1 MHz. The average power is 20 W above 50 kHz. The maximal energy-per-pulse, equal to 400 *μ*J, is available in the 0–50 kHz interval instead. Above these values, the energy per pulse is determined by the actual repetition rate setting: 200 *μ*J/pulse at 100 kHz, 100 *μ*J/pulse at 200 kHz, and 20 *μ*J/pulse at 1 MHz. Once the fundamental repetition rate is set, the corresponding energy/pulse is provided. From this condition, and using the laser-cavity Pockels cell, a lower repetition rate can be set by pulse-picking of the pulses; this possibility preserves the energy/pulse and is particularly useful for the optimization of non-linear optical processes since the same peak power is available but with a reduced thermal load. The repetition rate tuning flexibility has direct consequences on experiments, allowing us to find the ideal compromise between (probe) signal statistics and (pump-induced) sample heating. The laser source can seed two optical parametric amplifiers (OPA), configured for operation with 40 *μ*J/pulse or 360 *μ*J/pulse. They allow for a tunable output in the range 630–2500 nm and in the range 1350–4500 nm, which is also equipped with a difference frequency generation crystal to extend the output up to 16 *μ*m.

### B. HHG beamline

**FIG. 1.**
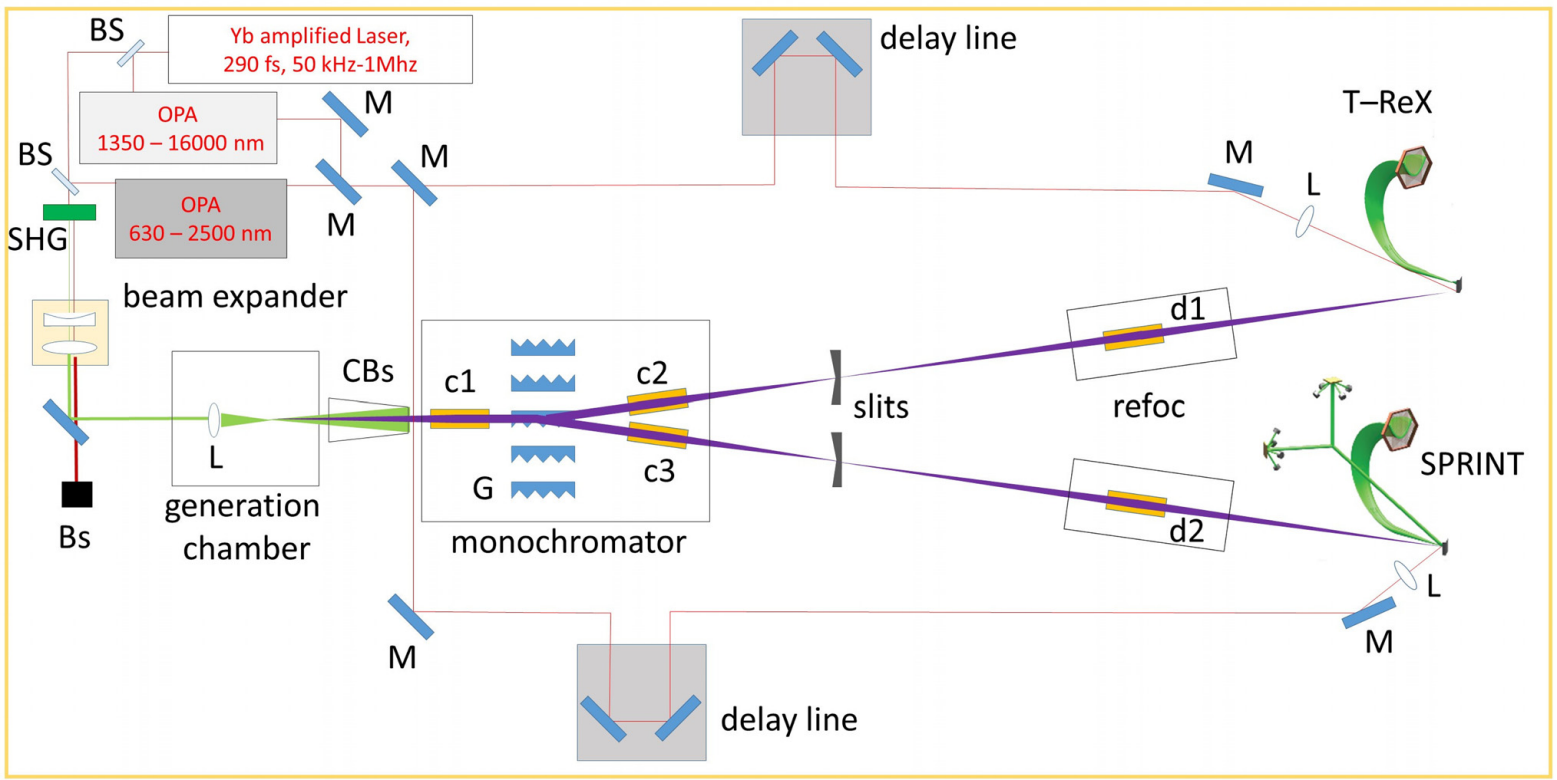
Pump/probe setup: M, mirror; BS, beam splitter; SHG, second-harmonic-generation crystal; Bs, beam stopper; CBs, cone beam stopper; L, lens; c1, c2, c3, d1, d2, toroidal focusing mirror; G, grating.

The HHG photon beam is propagated to two experimental end-stations designed for PES.^31,38^ The beamline is made up of a generation chamber, monochromator, and refocalization chamber, and coupled with OPA sources for time resolved pump-probe spectroscopy. The full optical path is reported in Fig. 1.

### C. Harmonic generation

The HHG process is a highly nonlinear optical effect emerging when the intensity of the laser light electric field is comparable to the atomic bond strength of a medium, most often a gas.^1^ This effect, known since more than 30 years, leads to the simultaneous generation of a number of odd harmonics of the seed photon energy, with almost constant intensity over a wide (plateau) energy region. It is usually described within the so-called three-step-model: tunnel ionization, free acceleration, and recombination (recollision).^39^

The generation chamber has been designed to work in the so-called tight-focusing regime^35^ that allows to reach the 10^14^ W/cm^2^ peak power density, required to drive the HHG process,^40,41^ also with a pulse duration of ∼300 fs.

In order to maintain a good vacuum level without overloading the main turbomolecular pump, a second chamber is installed around the gas nozzle, consisting in a sharp glass tip having 70 *μ*m internal diameter. This chamber is directly connected via an in-vacuum feedthrough to a 140 m^3^/h primary pump. The only apertures of the inner chamber toward the main chamber are two adjustable holes for the beam entrance and exit. This solution allows to routinely apply a gas pressure of several (4–6) bars at the gas nozzle input while maintaining a base pressure of $\approx 10^{-5}$ mbar in the main chamber. The gas nozzle is connected to a translation stage, which is used to optimize the nozzle position with respect to the laser beam.

In standard operating conditions, the HHG process is seeded by the second harmonics of the laser,^19,36,37^ at 515 nm. The frequency duplication of the laser radiation at 1030 nm is obtained with a 2 mm thick Beta Barium Borate (BBO) nonlinear crystal. The conversion efficiency into 515 nm radiation is 50% without beam focusing, giving 200 *μ*J/pulse at 50 kHz, 100 *μ*J/pulse at 100 kHz, and 50 *μ*J/pulse at 200 kHz after the crystal. Additional energy losses after the second-harmonic-generation (SHG) crystal, due to the optical elements along the beam path, reduce the energy per pulse in the generation chamber to 110 *μ*J/pulse at 50 kHz, 70 *μ*J/pulse at 100 kHz, and 29 *μ*J/pulse at 200 kHz.

The beam is then focused on the gas jet with a 10 cm focal length lens. We estimate the spot-size at the focus to be 10 ± 2 *μ*m. In order to dump the laser radiation used for generation, a cone-shaped beam stopper is inserted between the generation chamber and the monochromator. A hole of 1.5 mm on the vertex of the cone guarantees the complete transmission of the harmonics while limiting the seed beam transmission to only ≈ 1% of the input power. In this way, a longer lifetime of mirrors and gratings is expected. The beam dump also introduces a high vacuum impedance, reducing the pressure rise in the following vacuum chambers due to the generation gas. The cone is the only mechanical element introducing a differential pumping. The pressure increase in the UHV endstations is only marginally influenced by the gas. Usually the pressure in the UHV endstations rises from the base value of 1–3 × 10^–10^ mbar to 5–8 × 10^–10^ mbar when the valves to the refocusing chambers are opened. This happens irrespectively of the presence of the Ag gas used for HHG and mainly depends upon the fact that the base pressure in the refocusing chambers is slightly higher because of the presence of many motorized mirror actuators.

### D. Monochromator

The spectral selection of a single harmonics is performed by an off-plane-mount (OPM) grating monochromator. Differently from the classical diffraction, where the grating grooves (gr) are perpendicular to the incident plane, in the off-plane geometry the incident plane is almost parallel to the grooves. The main advantage of such a configuration is the capability to mitigate the EUV pulse-front tilt after diffraction, thereby providing a lower temporal broadening of the monochromatized pulses.^42^ Furthermore, the off-plane mount provides much higher EUV efficiency compared to the classical one.^43^

The instrument design consists of two toroidal gold coated mirrors and five OPM plane gratings (G in Fig. 1). The beam is collimated by a first toroidal mirror (c1) onto a specific grating and subsequently refocused onto one of the two exit slits by a second toroidal mirror (c2 or c3, depending on which branch is in use) with identical focal length in order to select a single harmonic beam and to steer it in either the SPRINT (Spin Polarized Research Instrument in the Nanoscale and Time) or T-ReX (Time Resolved X-Ray spectroscopy) end-stations.^44^ The mirrors are operated at equal grazing angle and unity magnification in order to minimize the aberrations at the output. The selection of a specific grating determines which end-station is served, without further adjustments. The specifications of mirrors and gratings are reported in Tables II and III, respectively. Considering the mirrors and gratings reflectivity, the entire system transmission, from the harmonics source to the sample, can be estimated around 46%. The grating parameters have been designed to introduce a dispersion sufficient to isolate a single harmonics with a slit width of ∼100 μm (adjacent harmonics are separated by 2.4 eV with 1030 nm seed and 4.8 eV with 515 nm seed). Since the intrinsic bandwidth of the harmonics is definitely lower than the bandwidth transmitted through the monochromator, the overall energy resolution is limited by the HHG source itself. Hence, the monochromator is acting as a tunable filter that is used to select a single harmonic and filter out all the adjacent harmonics.

**TABLE II.** Toroidal mirror specifications (*R_sag_*, sagittal radius; *R_tan_*, tangential radius; *f*, focal length; AOI, angle of incidence; reflectivity is estimated from the mirror specifications and AOI). c1, c2, and c3, toroidal mirrors in the monochromator chamber; d1, d2, toroidal mirrors in the refocalization chambers.

Mirror	*R_sag_* (mm)	*R_tan_* (mm)	*f* (mm)	AOI (°)	Coating	Reflectivity (%)
c1, c2, c3	41.9	8600	300	86	Gold	88
d1, d2	248	6813	1300	79	Gold	70

**TABLE III.** Grating specification; reflectivity is estimated from the grating specifications and AOI.

Grating	Blazing angle	Blazing energy	Range	AOI	Ruled area	Bandwidth	Reflectivity
(g/mm)	(°)	(eV)	(eV)	(°)	(mm)	(100 *μ*m slit) (eV)	(%)
150	3.4	18	8–30	5	70 × 10	0.5 at 20 eV	85
200	4.2	19	8–30	5	70 × 10	0.5 at 20 eV	85
400	4.5	36	30–50	5	70 × 10	1.1 at 40 eV	85
1200	7	70	50–100	5	70 × 10	1.45 at 20 eV	85

The use of gratings gives intrinsically a pulse-front tilt due to diffraction, which introduces a temporal broadening. The measure of the EUV divergence allows to estimate this broadening. The beam divergence has been measured through the knife-edge technique, resulting in 10 mrad for harmonics in the range 16.9 eV (i.e., 7th harmonic of 515 nm) to 31.2 eV (i.e., 13th harmonic of 515 nm). The resulting temporal broadening given by the monochromator because of the pulse-front tilt is in the range 50–100 fs, typically lower than the expected duration of the harmonics.

### E. Refocusing

The refocusing chamber is equipped with a toroidal mirror (d1 or d2, depending on which branch is in use; the specification are reported in Table II), which images the monochromatized EUV beam spot at the slits position onto the sample plane, with 1:1 ratio. A silver square mirror is placed in the refocusing chamber to direct the pump beam on the sample [m1 in Fig. 1(d), supplementary material]. The pump beam forms an approximately one degree angle with respect to the EUV probe pulse. The mirror is mounted on a piezo-mount to set precisely the pump-probe beam overlap. A second square mirror [m2 in Fig. 1(d), supplementary material], mounted off-center on a stepper motor, can be inserted in the beam path to send the quasi-collinear pump-probe beams out of the vacuum chamber (a 3D sketch of the beamline is reported in supplementary material). This possibility is used to optimize the beams focus (note that the pump beam is focused by a lens with focal length f = 1.5 m, placed before the entrance window on the refocusing chamber) and to roughly determine the time-zero condition. To this aim, the pump beam and the zero-order probe are sent on a fast photodiode recorded by a 4 GS/s oscilloscope. In this way, time-zero is pre-determined with a few ps uncertainty and finally found directly in the photoemission experiments.

## III. RESULTS

**FIG. 2.**
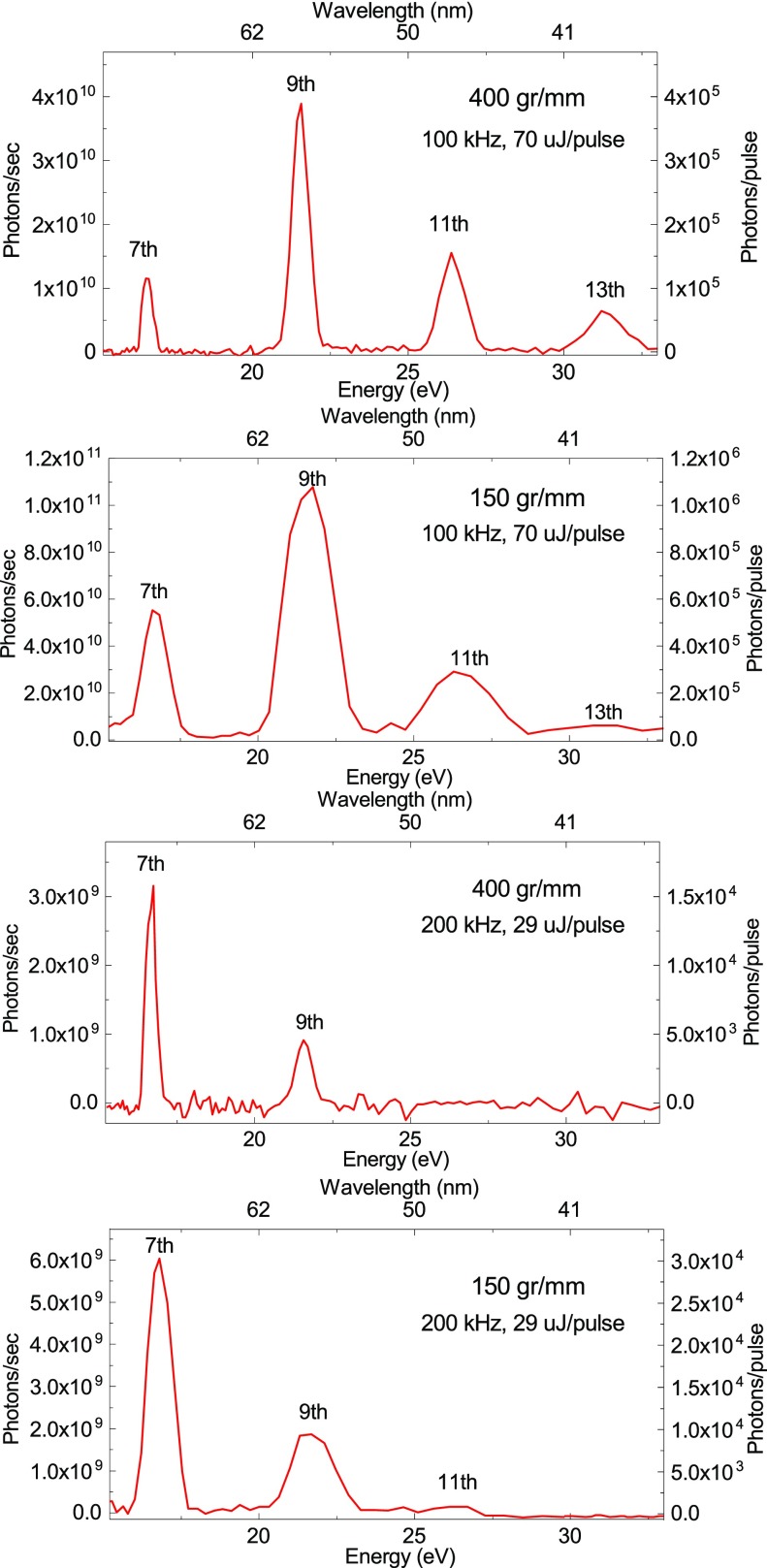
HHG spectra as resolved by gratings with 150 gr/mm and 400 gr/mm at a repetition rate of 100 and 200 kHz. All the spectra have been acquired with argon as the HHG medium, using a pressure of 4 bar at nozzle entrance. The identification of the harmonics is reported. The photons/pulse (photons/second) reported on the Y-axis is measured at the output of the monochromator, after passing the slit with an aperture of 100 *μ*m, using an x-ray photodiode, connected to an acquisition board. The pulse energy of the driving laser at 515 nm, measured into the generation chamber, is also reported in each panel.

Figure 2 reports the harmonics spectrum as measured by low energy gratings (150 and 400 gr/mm). Spectra were recorded under the same generation conditions, namely, the HHG process is driven by the SHG of the laser output, in argon (4 bar at nozzle entrance). The spectra have been measured by scanning the monochromator with 0.05° steps. The measurement of the photon flux at the output of the monochromator, after passing the slit with an aperture of 100  μm, has been performed with an x-ray photodiode (AXUV63HS1 from OPTO-DIODE Corp.), connected to an acquisition board. The conversion between current and number of photons has been obtained by using an analog photodiode calibrated at a synchrotron radiation facility. Each point has been acquired for 5 s. The measurements were performed at full power, at two different repetition rates. The number of photons per second measured in this condition is ≈ 0.4–1.1 × 10^11^ at 100 kHz and ≈ 1–6 × 10^9^ at 200 kHz.

Harmonics generation in neon with 1030 nm has been also tested, using a dedicated grating with 1200 gr/mm, generating up to the 63rd harmonics (not reported), with a two orders of magnitude lower photon flux.

**FIG. 3.**
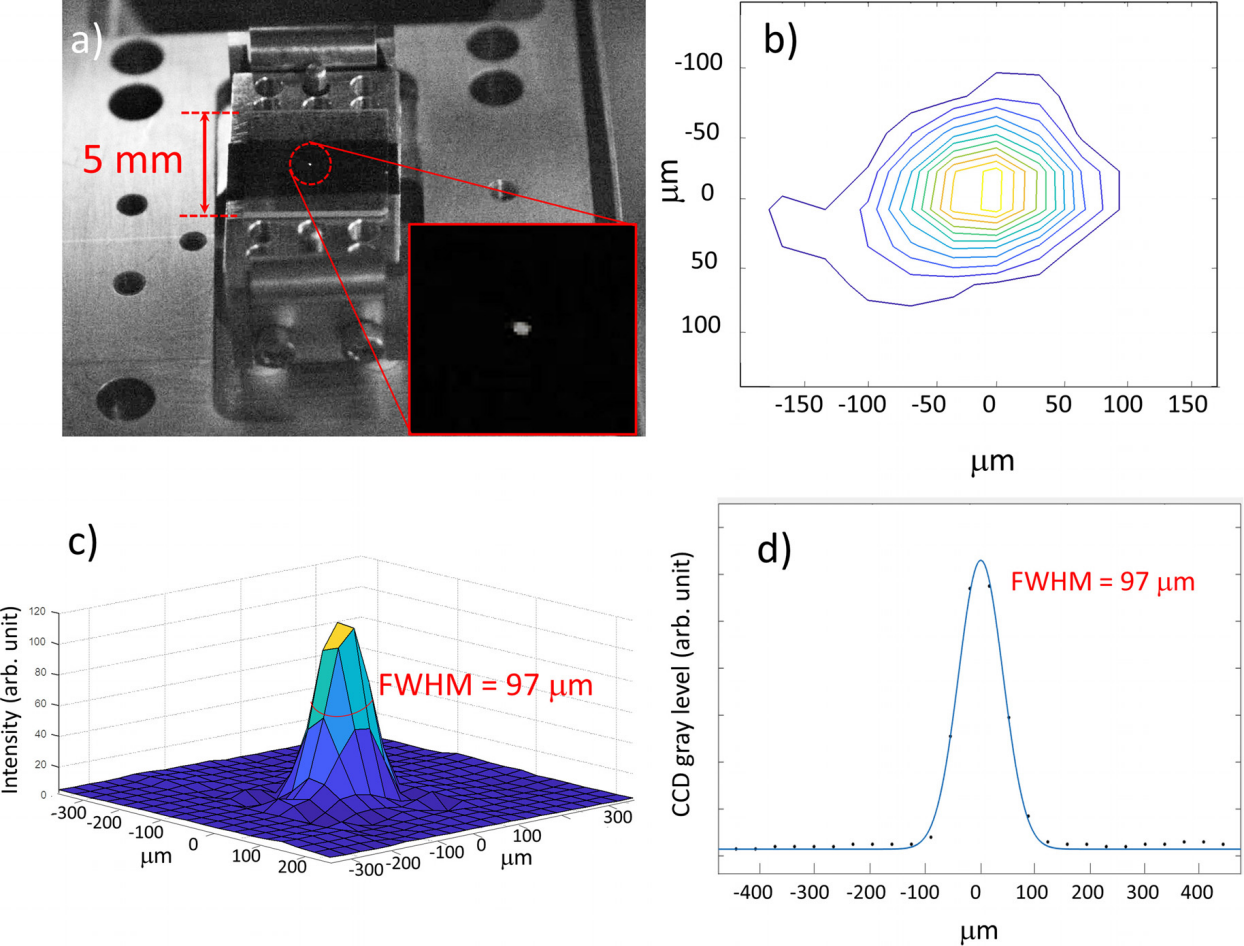
EUV focal spot. (a) Focal spot on the YAG screen; (b) contour plot of the focal spot; (c) 3D profile; (d) horizontal profile. The blue line represents the Gaussian fit.

The EUV focal spot is measured with a Yttrium Aluminum Garnet (YAG) crystal placed at the sample position. The EUV-beam fluorescence is recorded by a Charge-Coupled Device (CCD) camera. The horizontal profile of the EUV spot is fitted by a Gaussian function. The FWHM is 97 *μ*m, as shown in Fig. 3.

The HHG beamline feeds two end-stations optimized for complementary PES experiments: (a) The T-ReX end-station,^45^ hosting a SPECS electron analyzer equipped with a Delay Line Detector (DLD), optimized for time-resolved ARPES experiments. The T-ReX end-station features a six-degrees of freedom motorized cryomanipulator, which hosts the sample during experiments; details of the experimental setup can be found elsewhere;^31^ (b) the SPRINT end-station, where standard characterization techniques for surface science experiments (LEED, AES, ion bombardment) and a Scienta SES 2002 electron energy analyser (EA) are available.^38,46^ The experimental chamber also hosts a Vectorial Twin Mott detector setup^47^ for the measurement of spin polarization of the full or partial quantum yield from the sample,^38^ and a helium discharge lamp for reference spectra at 21.22 eV with 1 meV bandwidth and analyzer energy resolution calibration. Selected results obtained in commissioning experiments are reported in Secs. III A, III B, and III C.

### A. Space charge mitigation

When ultrashort UV pulses exceeding 10^5^ photons per pulse impinge on a sample surface, a large number of photoelectrons are created. The emitted electron cloud in front of the sample has the shape of a thin disk; hence, the electrons strongly interact with each other. This results in the fact that fast electrons are pushed ahead and slow electrons are retarded, producing an energy-shifted and momentum-distorted photoemission spectrum when more than one electron per laser shot is emitted.^48,49^ The typical strategies to mitigate space charge effects in photoemission are the reduction of the photon flux or the reduction of photon density on the sample surface by increasing the spot size. However, these workarounds are far from optimal as they lead to a reduction of the statistics of the measurement. This is a critical problem when dealing with low repetition rate pulsed UV or x-ray sources, like the present FEL (up to a hundred hertz) and the few kHz-range HHG sources.

The high repetition rate operation (up to 200 kHz) of our setup overcomes these problems. Indeed, high repetition rate operation makes it possible to maintain a high flux with a moderate intensity of the individual pulses, mitigating space charge.

The laser source used provides constant power (20 W) above 50 kHz operation. Hence one can reduce the energy per pulse available for HHG by increasing the repetition rate. However, due to the high nonlinearity of the generation process, the harmonic beam power is not constant. In addition, the harmonics energy cutoff is decreased. This sets an upper limit to the maximal repetition rate for each harmonics.

In order to demonstrate the possibility to mitigate space charge effects, we measured the valence band of a clean surface of a polycrystalline gold foil (Au displays a flat 6 s-like Density of State (DOS) across the Fermi level, suitable for the estimation of the energy broadening and shift induced by space charge) while tuning the generation conditions. In particular, we choose three different repetition rates (50 kHz, 100 kHz, 200 kHz), corresponding to three different energies per pulse at 515 nm into the generation chamber (see Sec. II C). The energy per pulse at fixed repetition rate can also be changed by an external attenuator (we report measurements down to 19.9 *μ*J at 200 kHz). It allows to analyze the evolution of the photoemission spectra as a function of the seed energy-per-pulse at a given repetition rate.

**FIG. 4.**
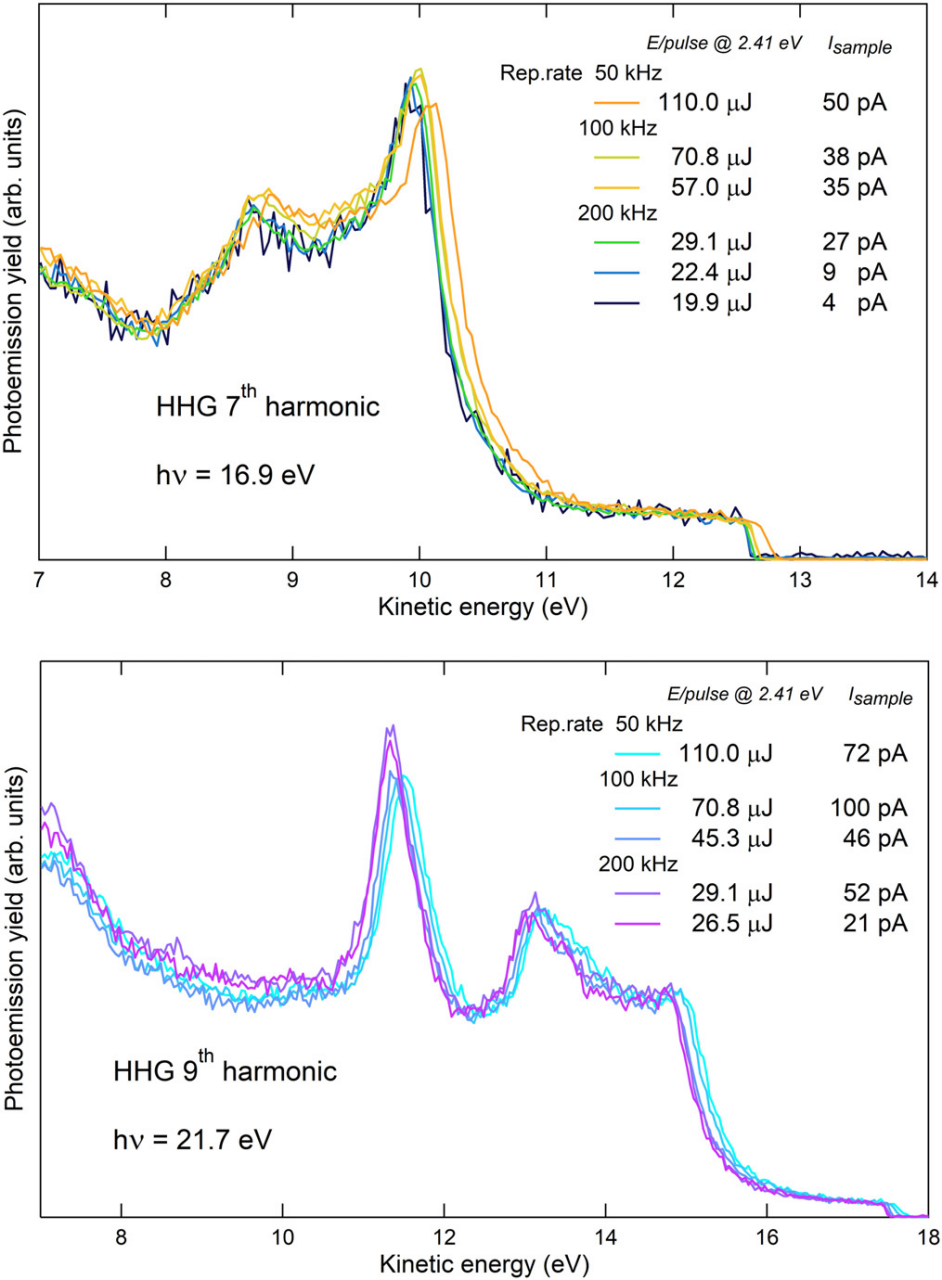
Angle integrated photoemission spectra of polycrystalline gold foil at T = 40 K, with 16.9 eV photon energy (7th HHG harmonic, upper panel) and 21.7 eV photon energy (9th HHG harmonic, lower panel), changing the energy per pulse of the driving laser from 110 *μ*J to 19.9 *μ*J; for each spectrum, the total electron yield (I_*sample*_) is reported as a measure of the total power impinging on the sample, with the correspondent total power impinging on the HHG gas nozzle. Spectra are rescaled so as to have the same height of the Fermi step.

**FIG. 5.**
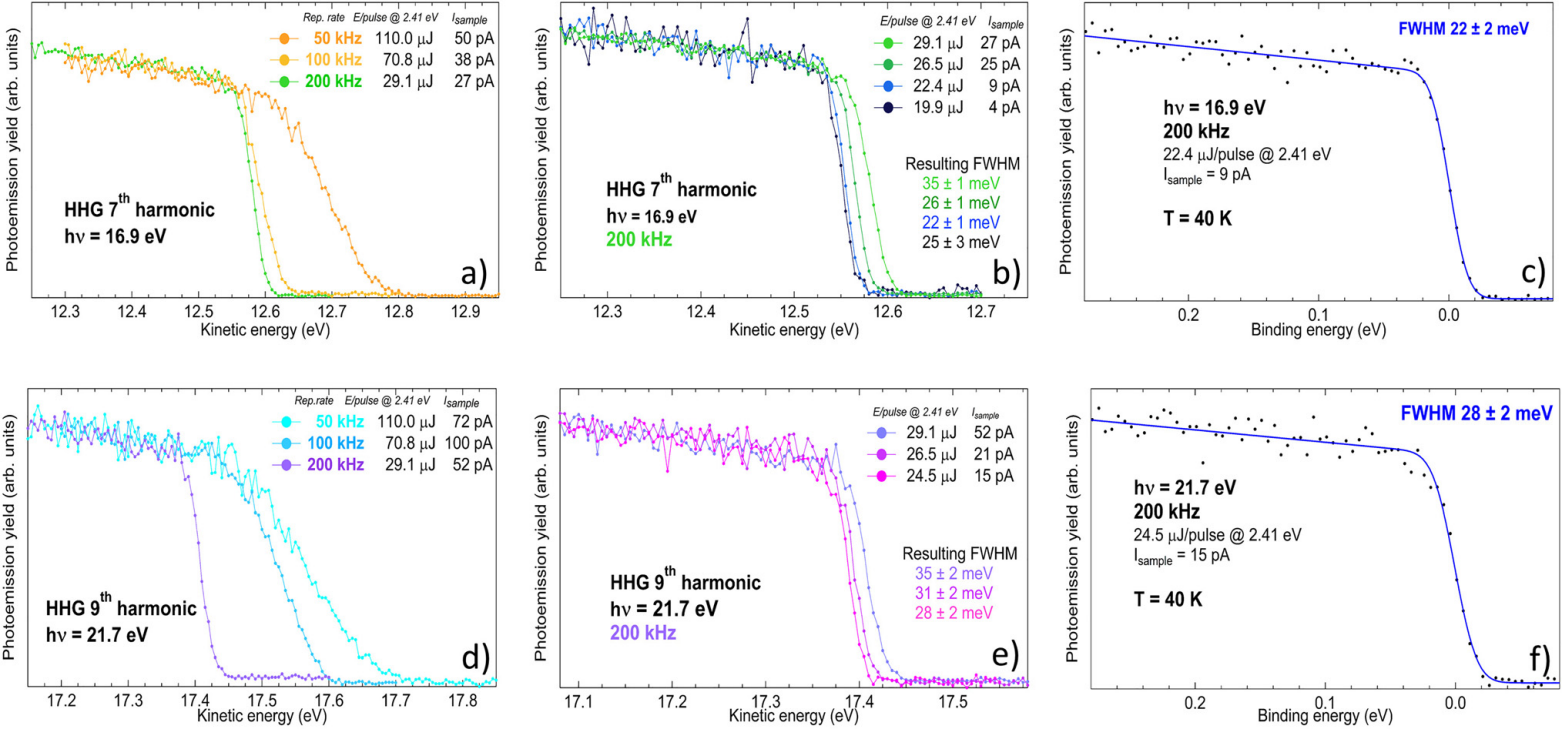
Fermi edge measured at 40 K for different laser repetition rates and photon energies. (a) Fermi edge at 16.9 eV (7th harmonic) measured at 50, 100, and 200 kHz repetition rate; (b) Fermi edge at 16.9 eV, 200 kHz, varying the energy per pulse of the driving laser; (c) 7th harmonic energy bandwidth measurement. Square black dots: experimental data. Solid blue line: fitting curve used to extract the FWHM; (d) Fermi edge at 21.7 eV (9th harmonic) measured at 50, 100, 200 kHz repetition rate; (e) Fermi edge at 21.7 eV, 200 kHz, varying the energy per pulse of the driving laser; (f) 9th harmonic energy bandwidth measurement.

The angle integrated photoemission spectra obtained with the 7th and the 9th harmonic are displayed in Fig. 4 as a function of the energy per pulse of the driving laser. The spectra are measured at a temperature T = 40 K. For each spectrum, the total electron yield is measured by recording the drain current with a picoammeter (Keithley 6482), which connects the sample to the electrical ground. As the amount of space charge is reduced, the spectra shifts toward lower kinetic energy and the Fermi edge (E_*F*_) becomes steeper. This feature is showed in detail in Fig. 5. Panel (a) shows a zoom of the Fermi edge on gold measured with the 7th harmonic, changing the repetition rate from 50 kHz (orange curve) to 200 kHz (green curve): a strong reduction of the broadening and the shift of the Fermi step is achieved.

Panel (b) shows the spectra measured at 200 kHz upon further reducing the energy-per-pulse by means of attenuation of the driving laser: some very small space charge effects are actually still present at maximum fluence (light green curve), and the space charge is totally removed when the total electron yield is below 20 pA (blue and black curves), corresponding to around 600 photoelectrons per pulse. Panels (d) and (e) show the spectra measured with the 9th harmonic, where a similar trend can be observed.

### B. Harmonics energy bandwidth

Once the space-charge related effects have been controlled, a reliable measurement of the best attainable energy resolution can be performed by investigating the Fermi edge of polycrystalline Au at low temperature.

The Fermi edges measured with the 7th harmonic at 16.9 eV and the 9th harmonic at 21.7 eV at the SPRINT end-station (analyzer pass energy 5 eV, entrance slit 0.5 mm) are displayed in Fig. 5 panels (c) and (f), respectively. The fitting curve (blue line) is a convolution of the Fermi function $f(E_{K})={\left(e^{\frac{E_{K}-E_{F}}{k_{B}T}}+1\right)}^{-1}$, with a Gaussian, accounting for the instrumental resolution, including the source photon bandwidth. While the temperature is fixed and known by independent measurement, the free parameters of the fit are *E_F_* and the FWHM of the Gaussian itself, and the slope and intercept of the line mimicking the DOS. The resulting FWHM is 22 ± 2 meV at 16.9 eV and 28 ± 2 meV at 21.7 eV. This value can be decomposed as the sum in quadrature of the source and the detector contributions,^50^ as well as other instrumental broadenings (electronic noise): $\mathrm{FWH}M^{2}=△E_{\mathrm{source}}^{2}+△E_{\mathrm{detector}}^{2}+△E_{\mathrm{other}}^{2}$ According to the formula $△E_{\mathrm{detector}}=(W/2R_{0})E_{p}$ (W = 0.5 mm, R_0_ = 200 mm, E_*p*_ = 5 eV), the expected value of the detector resolution in the configuration of this measurement is 6.25 meV (to this value one should add the term $\alpha^{2}/4E_{p}$ which accounts for the angular spread *α* of the electrons transmitted into the hemispheres).^51^ We have evaluated directly the detector resolution by measuring the Fermi edge at liquid nitrogen temperature with a known source (He I lamp, 21.22 eV) as a function of the pass energy, yielding $△E_{\mathrm{detector}}=(0.0019\pm 0.0006)\times E_{P}$; since in our case *Ep *=* *5 eV, the result is $△E_{\mathrm{detector}}=9.5\pm 3.0$ meV, which is in good agreement with the expected value, considering the presence of the angular term. Such a value is negligible with respect to the total FHWM retrieved by the fit in Fig. 5, panels (c) and (f). Hence, we conclude that the reported values are the upper limit for the overall energy resolution measured with the 7th and 9th harmonic. Most importantly, in Fig. 5, panels (b) and (e), we report the overall energy resolution obtained with the presence of space charge, for both the harmonics at 200 kHz. Also, in the worst condition, the overall energy resolution never exceeds 35 meV.

### C. Harmonics duration

**FIG. 6.**
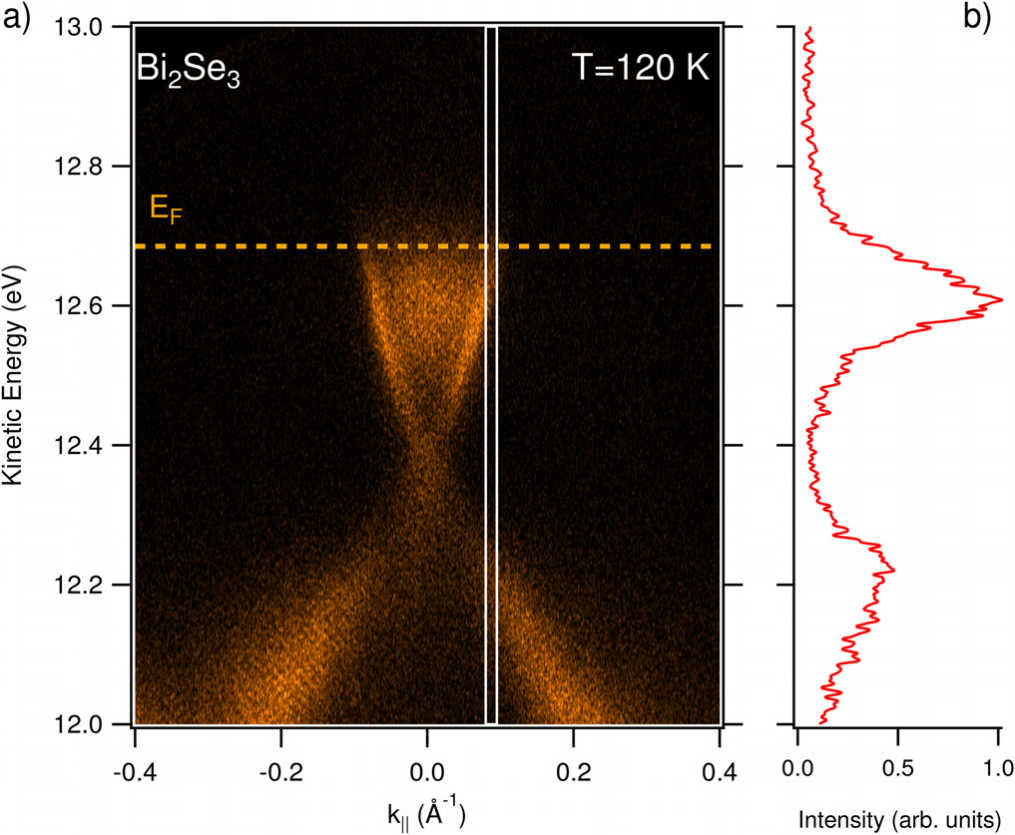
ARPES experiment. (a) ARPES map acquired on bismuth selenide at T = 120 K, and hν = 16.9 eV (7th harmonic). (b) EDC profile integrated from the white box (0.015 ${Å}^{-1}$ wide) drawn on panel (a).

**FIG. 7.**
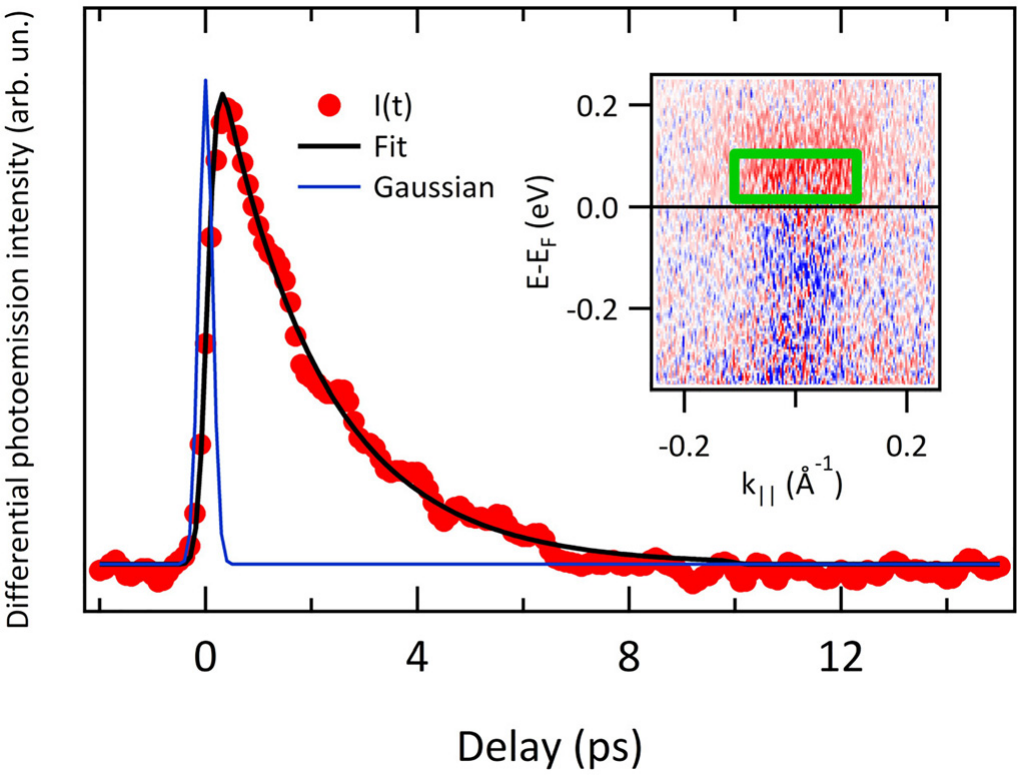
Electron dynamics acquired in the pump-probe experiment. Photoemission intensity has been averaged in the green box overlaid on the ARPES map reported as the inset, which shows the differential ARPES intensity collected at the delay t = 300 fs. The black line in the main panel is the fit to the data (see main text for details). Blue line is the Gaussian representing the pump-probe cross correlation, as retrieved by the fitting routine.

In this section, we report on the results obtained by ARPES at equilibrium and out-of-equilibrium, using the EUV high-harmonics as a probe. The experiments have been performed at the T-ReX end-station equipped with a SPECS Phoibos 225 hemispherical electron analyzer, using the topological insulator Bismuth Selenide (Bi_2_Se_3_, provided by HQ Graphene) as a reference sample. The sample temperature was set to T = 120 K. Figure 6(a) shows the ARPES map acquired on the Bi_2_Se_3_ sample in s-polarization, using the 7th harmonic (h*ν* = 16.9 eV) as a probe. The pass-energy of the analyzer was set to 15 eV (the entrance-slit width is 0.5 mm), and the repetition rate of the laser source was 100 kHz (set for reducing the average pump power). The total acquisition time was ≈ 10 min, working with ≈ 3 × 10^8^ photons/s, corresponding to a regime of moderate-flux. Indeed, the intensity of the EUV harmonics has been attenuated to avoid space charge effects by reducing the intensity of the seeding laser pulse. Under these conditions, space-charge is minimized, and a reasonable count rate is achieved, as it is appreciated from the quality of the ARPES map reported in Fig. 6(a). Figure 6(b) shows an energy profile extracted from the region highlighted in panel (a); the broadening of the profile is due to the large momentum integration window, chosen to reduce the noise. The out-of-equilibrium experiment was performed under the same experimental conditions, the only difference being the analyzer slit width, which was set to 2 mm to reduce the acquisition time. As a pump, we used the fundamental of the laser source at 1030 nm. The beam was focused on the sample through a f = 1.5 m focal length lens, down to a spot size of 300 ± 10 *μ*m. The pump-probe spatial overlap was set by superimposing the two beams on a cerium-doped YAG scintillator that converts EUV radiation in visible photons (at ≈ 560 nm) that are imaged by a complementary metal-oxide semiconductor (CMOS) camera. In the experiment, the pump fluence was set to 150 ± 30 *μ*J/cm^2^. The results of the pump-probe experiment are reported in Fig. 7. In the pump-probe scan, the step of the delay-line was set to 66.67 fs, which is below the expected time resolution. The integration time for data reported in Fig. 7 is 2 h. The inset of Fig. 7 shows the differential ARPES map computed by subtracting a map acquired before excitation (t = −500 fs) from the map acquired at t = +300 fs. In this way, the effect of the pump excitation is evident. The trace reported in Fig. 7 shows the electron dynamics extracted by averaging the ARPES intensity in the green box overlaid to the ARPES map reported as the inset. The electron dynamics has been analyzed with a single exponential decay convoluted to a Gaussian (reported in Fig. 7 as a solid blue line) representing the pump-probe cross correlation. The only free parameters of the fit (solid black line) are the intensity I and time-constant *τ* of the exponential decay, and the Gaussian FWHM, *σ*. We obtain τ=2±0.1 ps and σ=300±30 fs. The duration of the pump-pulse has been measured independently with an auto-correlator (APE Berlin PulseCheck) in the last portion of the pump beam path, which resulted as $\sigma_{\mathrm{pump}}=280\pm 5$ fs. With this value, we determine an upper limit for the XUV probe FWHM after deconvolution of *σ_pump_* from *σ*. The result is $\sigma_{\mathrm{probe}}=105\pm 45$ fs, which is reasonable if we consider the natural pulse shortening obtained both in the SHG process (the FWHM of the 515 nm seed beam is 230 ± 5 fs, as measured with the auto-correlator) and in the subsequent HHG process. The value we provide constitutes an upper limit for the harmonics duration since a non-negligible rise time in the time-resolved ARPES signal measured on Bi_2_Se_3_ is likely present. For Gaussian pulses, the minimum bandwidth required to sustain a pulse-duration of 105 fs is ≈ 17 meV. By comparing this value with the upper limit of the energy bandwidth as determined by photoemission measurements, we can conclude that we are close to the transform-limit condition.

## IV. CONCLUSIONS

In this work, we have addressed a major issue in time-resolved PES by mitigating the space charge effect that significantly depletes the overall energy and momentum resolutions.

Here, we demonstrate that with a remote tuning of the repetition rate up to 200 kHz, hence reducing the number of photons/pulse (≈5 × 10^8^ corresponding to ≈ 3 × 10^7^ electrons/s), it is possible to perform space-charge free photoemission with ultrashort EUV (17–31 eV) photon pulses.

From the momentum-integrated Fermi edge of polycrystalline Au at 40 K, we retrieved an ultimate overall energy resolution of ∼22 meV at 16.9 eV. This resolution, close to the HHG pulse bandwidth of 19 meV, when compared with a photon pulse duration of ∼105 fs, demonstrates that time resolved PES experiments can be performed under Fourier transform limit conditions.

As a consequence, the laser source, the high-harmonic-generation, and the photon beam optics, as reported in this work, open the way to time-resolved PES experiments where the optimal trade-off between time and energy resolution is achieved, while the full Brillouin zone of crystalline solids can be accessed and a wide tunability of the repetition rate is allowed.

## SUPPLEMENTARY MATERIAL

See the supplementary material for a detailed description of the HHG beamline.
